# Effects of PPA Reinforcement and Sintering Parameters on the Densification and Hardness Properties of Al/Mg/PPA Composites

**DOI:** 10.3390/ma18061246

**Published:** 2025-03-11

**Authors:** Osarue Osaruene Edosa, Francis Kunzi Tekweme, Peter A. Olubambi, Kapil Gupta

**Affiliations:** 1 Department of Mechanical and Industrial Engineering Technology, University of Johannesburg, Johannesburg 2028, South Africa; osaruene@gmail.com (O.O.E.); ftekweme@uj.ac.za (F.K.T.); 2 Centre for Nanoengineering and Advanced Materials, School of Mining, Metallurgy and Chemical Engineering, University of Johannesburg, Johannesburg 2028, South Africa; polubambi@uj.ac.za

**Keywords:** composites, hardness, relative density, agro-waste ash, SEM, sintering, XRD

## Abstract

The utilization of agricultural wastes in composite fabrication leads to attaining sustainability in manufacturing. This study investigates the use of plantain peel ash (PPA) as a reinforcement to fabricate Al/Mg/PPA composites using ball milling and spark plasma sintering (SPS) technology. The impact of PPA weight fraction and SPS parameters on Al/Mg/PPA composites’ densification and hardness were analyzed. Microstructural characterization revealed that the PPA reinforcement was uniformly distributed in the aluminum matrix with no considerable microstructural defects. The relative densities of the composites were higher at elevated sintering temperatures, with composites displaying reduced porosity as the sintering temperature rose. The composites also exhibited the highest micro-hardness of 77 HV, improving 86.89% over the sintered aluminum matrix (base material). The Analysis of Variance (ANOVA) results revealed that the sintering temperature and reinforcement material significantly influenced the relative density (RD) of the sintered composites, while the reinforcement material significantly influenced the micro-hardness. Conclusively, the composite samples made using agricultural waste derivatives possess good mechanical properties and are suitable for various industrial applications.

## 1. Introduction

Agricultural waste reinforcements have continued to be the focus of research in aluminum matrix composite (AMC) because of their potential to enhance composites’ physical, mechanical, and tribological properties [1,2,3]. As reported in the literature, synthetic and agro-industrial waste derivatives are the two main types of ceramic reinforcements [3,4]. Commonly used synthetic reinforcements include SiC, Al_2_O_3_, AlB_2_, B_4_C, MgO, TiC, WC, and TiB_2_ [2,5], while commonly used agro-industrial waste reinforcements include fly ash, rice husk ash, palm kernel shell ash, wheat husk ash, corn cob ash, sugarcane bagasse, and bamboo leaf ash [4,6,7].

The types of reinforcements used and the processing methods significantly impact the quality and performance of composites. As an alternative to synthetic reinforcement, agricultural waste reinforcement has gained much interest [3,8]. The high acquisition cost and limited availability are some of the drawbacks of synthetic reinforcements.

Using agricultural waste as reinforcement is especially important for promoting a circular economy, which will positively impact the environment by reducing waste and pollution and using fewer natural resources. This is especially true when it comes to achieving zero-waste-to-landfill goals [9]. The percentage chemical composition of agricultural wastes (Al_2_O_3_, MgO, SiO_2_, K_2_O, ZnO, MnO, Fe_2_O_3_, CaO, P_2_O_5_, and TiO_2_), which varies from plant to plant, can be linked to their performance as reinforcements. According to previous studies, AMC’s microstructure and properties significantly improved due to the performance of agricultural waste reinforcements [5,10]. Agricultural waste reinforcements have lower economic costs and density advantages. One intriguing feature of agricultural waste ashes is that their percentage chemical composition changes with heat treatment temperatures. For instance, Abshalomu et al. [10] found that when the heat treatment temperature increased from 400 to 600 °C, the percentage concentration of SiO_2_ in cashew nut ash increased from 44 ± 2% to 75 ± 2%. Using agricultural waste derivatives as the only reinforcement presents several challenges, including reduced strength, ductility, and fracture toughness [3,8]. However, a significant improvement was seen when agricultural waste reinforcements were combined with synthetic reinforcements [8,11]. It has been proposed that agricultural waste reinforcements supplement synthetic reinforcements because of their inadequacy. Therefore, combining agricultural waste reinforcements with synthetic reinforcements is one efficient way to use them to synthesize AMC [11,12].

The properties of the aluminum matrix and reinforcement define the attributes of the AMC. The properties of AMC depend on the concentration and characteristics (the chemical composition, size, and shape) of the reinforcement. The automotive and aerospace industries, among others, have found it necessary to employ AMC due to its appealing qualities. When employed, AMC has excellent performance and fuel efficiency, among other advantages [13]. AMC reinforced with agricultural waste also has benefits such as low density and coefficient of thermal expansion. The sustainability criteria for any synthesized material include affordability, environmental friendliness, and societal advantages [14]. The uninterrupted fabrication of sustainable AMC will depend on the constant availability of reinforcement materials to avoid supply shortages due to the growing demand for AMC’s products. Utilizing green and renewable agricultural waste derivatives as reinforcement materials is one approach to assure sustainability in the manufacturing of AMC. Ikele et al. [12] fabricated hybrid reinforced AMC using SiC and palm kernel shell ash (PKSA). According to the authors, the best value of the mechanical property of the composites was obtained when the weight ratio of SiC to PKSA reinforcements was 1:1, indicating that agricultural waste reinforcement is a great addition to synthetic reinforcement.

Another factor that may affect the quality of AMC is the process of fabrication. There have been several documented methods for producing AMC [3,15,16]. The two most frequently used methods are powder metallurgy (PM) and stir casting. This study explores the capability of the PM process in producing high-quality AMC. The main objective of PM technology for fabricating AMC has been to improve the densification and microstructural features, which ultimately enhances the mechanical properties such as hardness, strength, and fracture toughness of the composites [15,17,18]. In this study, AMC reinforced with plantain peel ash is fabricated using ball milling and spark plasma sintering (SPS). A detailed analysis and discussion of the effects of ball milling parameters, sintering parameters, and reinforcement concentration on the fabricated AMC’s microstructure, densification, and hardness have been conducted.

## 2. Materials and Methods

### 2.1. Starting Materials

**Figure 1:**
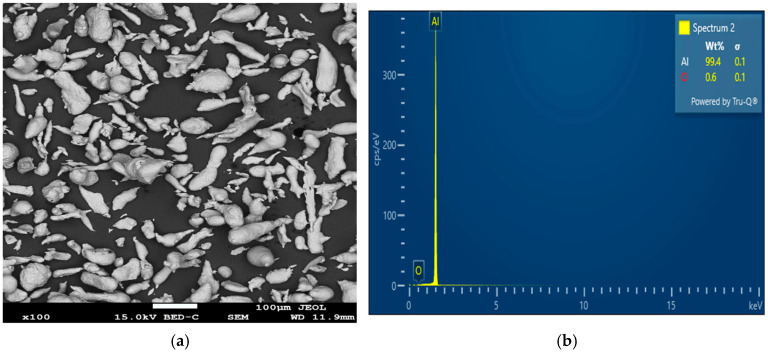
(**a**) SEM image; (**b**) SEM-EDS analysis of pure aluminum powder particles.

The starting materials for synthesizing the AMC samples for this investigation include pure Al powder (75 µm), Plantain peel ash (70 µm), and Mg powder (100 µm). The Al matrix, purchased from Zimco Aluminum Company, Benoni, South Africa, was reinforced with 0, 5, 10, 15, and 20 wt% PPA. The 2 wt% Mg powder, purchased from Sigma-Aldrich, Johannesburg, South Africa, was introduced into the Al/PPA mixes to improve the wettability [19]. The SEM image and SEM-EDS analysis of the pure Al particles are shown in Figure 1. From the figure, it can be seen that mixed sizes, oval, irregular, and flaky shapes characterize the aluminum powder particles. The processing of the plantain peel wastes to obtain ash is presented in Section 2.2.

### 2.2. Preparation of Plantain Peel Ash Reinforcement

**Figure 2:**
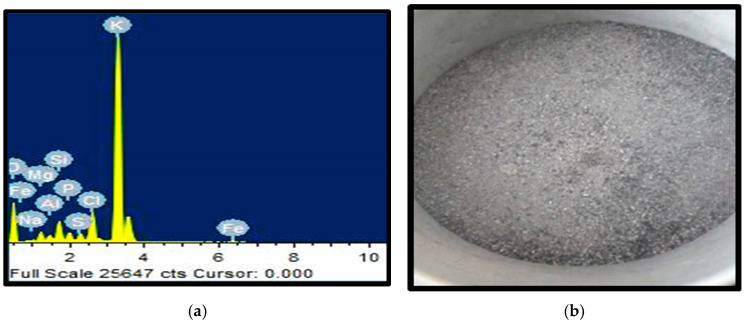
Plantain peel ash (**a**) EDS analysis, (**b**) powder particles.

The plantain peel wastes were sourced from Harper, Liberia, and processed to obtain ash following the procedure described in the literature [8,12]. The processing stages include: (i) sourcing the wastes and drying them in the sun for about two weeks to reduce the moisture content; (ii) burning the dried plantain peel in an incinerator to remove organic material and produce ash residue; (iii) heat treating the ash residue at an average temperature of 550 °C using a furnace to remove the black carbon and other volatile materials. This makes it fit for use as ceramic reinforcement, see Figure 2b. The heat-treated ash was then pulverized and sieved to obtain fine powder particles. Characterization of the ash was done using an SEM-EDS machine (JEOL JSM-7900F, JEOL, Peabody, MA, USA) to determine its elemental composition. The result obtained includes 79.42% K, 4.70% Fe, 4.46% P, 4.18% Si, 1.45% Al, and 1.26% Mg, as illustrated in Figure 2a. The density of the PPA was determined to be 2.34 g/cm^3^ using a gas pycnometer (BetterPyc 380, Bettersize, Noi, Vietnam).

### 2.3. Design of Experiment Using the Taguchi Method 

The Taguchi technique is selected to design the experiments in the present study. It uses Orthogonal Arrays (OAs) to systematically vary and test each parameter at different levels (intervals). The orthogonal array provides a set of well-balanced control parameters with settings to achieve the best performance characteristics. The control parameters for this study include reinforcement weight fraction, sintering temperature, sintering pressure, heating rate, and soaking time. The design of experiments for this study uses (L_25_5^5^) Taguchi orthogonal array with five parameters tested at five levels, resulting in 25 experimental runs (samples).

### 2.4. Al/Mg/PPA Composite Sample Production

**Figure 3:**
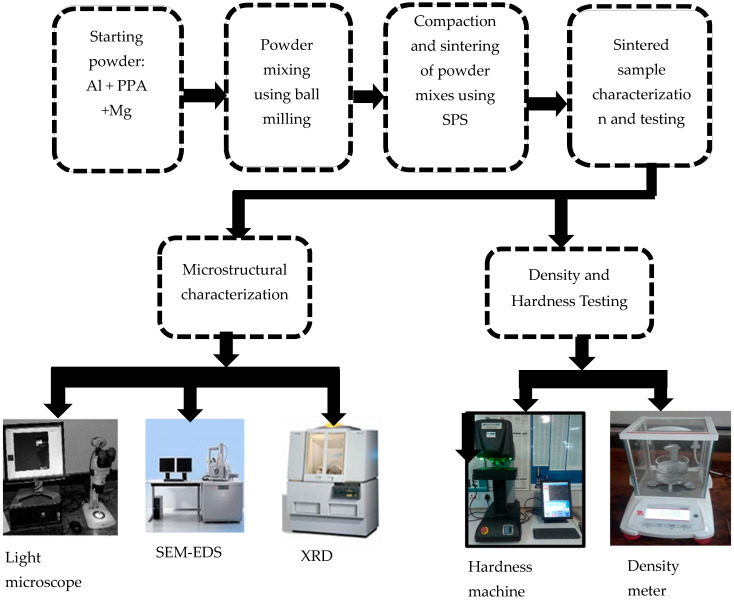
The flow chart for the fabrication of Al/Mg/PPA composites.

Al/Mg/PPA samples were fabricated using the PM method (Ball milling and SPS). The first stage in the processing sequence of the PM process involves powder mixing using a high-energy ball mill (PM 100, Retsch, Haan, Germany). The powder mixes were made according to the ratio presented in Table 1 and charged in a chromium steel vial with a ball-to-powder ratio (BPR) of 5:1 under normal atmospheric conditions. The powder mixtures were milled for 90 min at an average speed of 200 rpm. To prevent excessive cold welding and powder particle agglomeration, 1 wt% of propanol was added to the powder mixture. After every 30 min of milling, the milling machine was turned off for 10 min to prevent the milling vial’s temperature from rising [18,20]. The compaction and sintering of the milled powders were done using the SPS machine (8604 HHPD-25, FCT Systeme GmbH, Frankenblick, Germany), having a graphite die, a maximum compaction force of 250 KN, a working temperature of 2200 °C, a maximum sintering current of 8000 A, and a vacuum atmosphere. The die walls were covered with graphite sheets before filling with nine grams of the powder mixture. The graphite sheet prevents the sintered samples from sticking to the die walls, thereby facilitating their removal from the die. After the sintering time was completed, the sintering current was switched off to allow the samples to cool to room temperature. The graphite sheets, which formed additional layers to the sintered samples, were removed using a knife. The dimensions of the fabricated composites after the graphite sheet layers have been removed are 20 mm in diameter and 12 mm in thickness. The sintering parameters for this study are presented in Table 2. Figure 3 illustrates the flow chart for the fabrication of Al/Mg/PPA Composites.

### 2.5. Characterization of Samples

The microstructure of the fabricated Al/Mg/PPA samples was characterized using XRD (Empyrean, Worcestershire, UK), light microscope (ZEISS, Jena, Germany), and SEM-EDS, following the standard procedure [19]. Sample preparation was by grinding and polishing using abrasive papers and polishing cloths mounted on the grinding and polishing machine (ATM Saphir 500, ATM Qness GmbH, Mammelzen, Germany) to have a smooth and mirror-like surface finish. Etching of each sample to make the grain boundaries more visible took 30 s using Keller reagent (190 mL distilled water + 5 mL HNO_3_ + 3 mL HCl + 2 mL HF). The etching process was consecutively halted by immersing the samples in alcohol and distilled water.

### 2.6. Density Measurement

The densities of the developed Al/Mg/PPA samples were determined using a digital density meter under ambient temperature and pressure, following the ASTM B962-13 standard, according to Archimedes principles. The density meter has an accuracy of 0.0002 g/cm^3^ and an average value of five measurements was reported. The density ($\rho_{c})$, theoretical density ($\rho_{th})$, relative density (RD), and the porosity of Al/Mg/PPA samples were determined using Equations (1)–(4).

(1) $$\rho_{c}=\frac{w_{a}}{w_{a}-w_{w}}\times \rho_{w}$$

(2) $$\rho_{th}=\rho_{Al}{wt}_{Al}+\rho_{Mg\;}{wt}_{Mg}+\rho_{PPA}{wt}_{PPA}$$

(3) $$RD=\frac{\rho_{a}}{\rho_{th}}\times 100$$

(4) $$Porosity\;\left(\emptyset \right)=1-\frac{\rho_{a}}{\rho_{th}}$$

where $\rho_{c}$ = density of the fabricated composites,$\;w_{a}$ = weight in the air, $w_{w}$ = weight in water, $\rho_{w}$ = density of water, $\rho_{th}$ is the theoretical density of the composites, ρ_Al_ = theoretical density of the Al matrix (2.7 g/cm^3^), wt_Al_ = Weight fraction of Al matrix, $\rho_{Mg\;}$= theoretical density of the Mg (1.74 g/cm^3^), ${wt}_{Mg}$ = weight fraction of magnesium, ρ_PPA_ = theoretical density of the PPA reinforcement (2.34 g/cm^3^), wt_PPA_ = weight fraction of PPA, and RD = relative density.

Applying the rule of mixture expressed in Equation (2), the evaluated theoretical densities of the fabricated Al/Mg/PPA samples are presented in Table 1.

### 2.7. Hardness Testing

Hardness testing is a non-destructive test carried out by applying a constant load through a diamond indenter on the polished surface of the specimen to create an indentation on the surface, which is then measured to determine hardness. The hardness of the developed Al/Mg/PPA samples was tested following ASTM E 92 Standard using a Vickers’ hardness testing machine with a capacity of 100 kgf (Innovatest Falcon 500, Innovatest, Maastricht, The Netherland, equipped with a diamond pyramid indenter). A constant load of 100 gf was applied for 10 s to the polished sample surfaces, and an average value of five trials was recorded. The RD, hardness, and standard deviation for hardness of the sintered samples are presented in Table 3.

## 3. Results and Discussion

### 3.1. Optimal Input Parameters for RD

**Figure 4:**
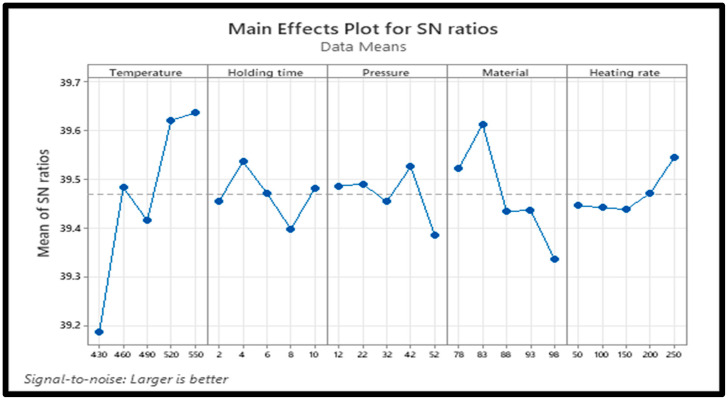
Main effect plot of signal-to-noise ratios for RD.

Optimizing the processing parameters for RD results in the best value of output. Using Minitab 20 and the larger-the-better approach expressed in Equation (5), the results of optimization are presented in Table 4 and Table 5, and Figure 4, respectively.

(5) $${SN}_{L}=-10log(\frac{1}{n}\sum_{i=1}^{n}\frac{1}{y_{i}^{2}})$$

where y_i_ = response, n = number of trials for y_i_.

The results of the Taguchi analysis presented in Table 4 and Table 5, and Figure 4 show that the sintering temperature strongly impacted RD (rank 1), followed by the PPA reinforcement (rank 2). The heating rate had the least effect on the RD. As illustrated in Figure 4, the optimal processing parameters for the RD of Al/Mg/PPA samples include a sintering temperature = 550 °C, holding time = 4 min, compaction pressure = 42 MPa, PPA concentration = 15 wt% PPA, and a heating rate = 250 °C. The effects of each input parameter on the RD and their significance level were determined using ANOVA.

As illustrated in Table 5, the F-value and *p*-value show the factor’s significance level. As indicated by the F-value column, the factor with the highest F-value has the strongest influence and vice versa. Similarly, from the *p*-value column, factors having values less than 0.05 (*p* < 0.05) are statistically significant. As shown in Table 5, temperature and material are the only parameters that have a statistically significant influence on RD. The remaining parameters, though, contributed to the enhanced densification of the composites but in a smaller magnitude.

### 3.2. Influence of Input Parameters and Microstructure on Relative Density (RD) and Porosity

**Figure 5:**
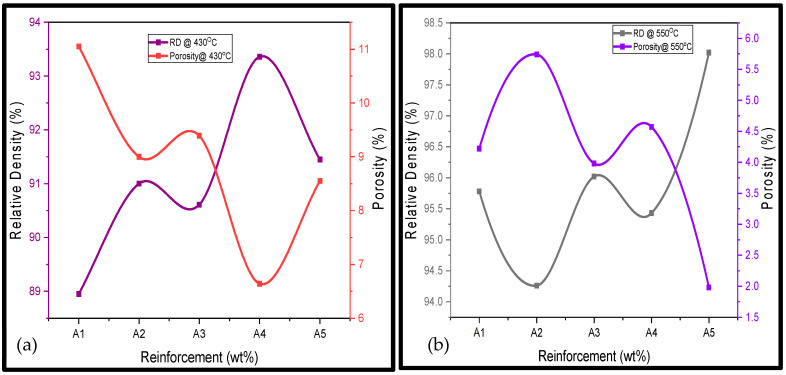
Relationship between RD and porosity of Al/Mg/PPA composites sintered at (**a**) 430 °C and (**b**) 550 °C.

The porosities of the fabricated Al/Mg/PPA composites were estimated using Equation (4). Porosity in composites can be reduced but cannot be eliminated. As the RD decreases, the porosity of the Al/Mg/PPA composites rises, as illustrated in Figure 5. The main factors influencing the porosity levels of the fabricated composites include the reinforcement characteristics and the processing parameters [21,22].

**Figure 6:**
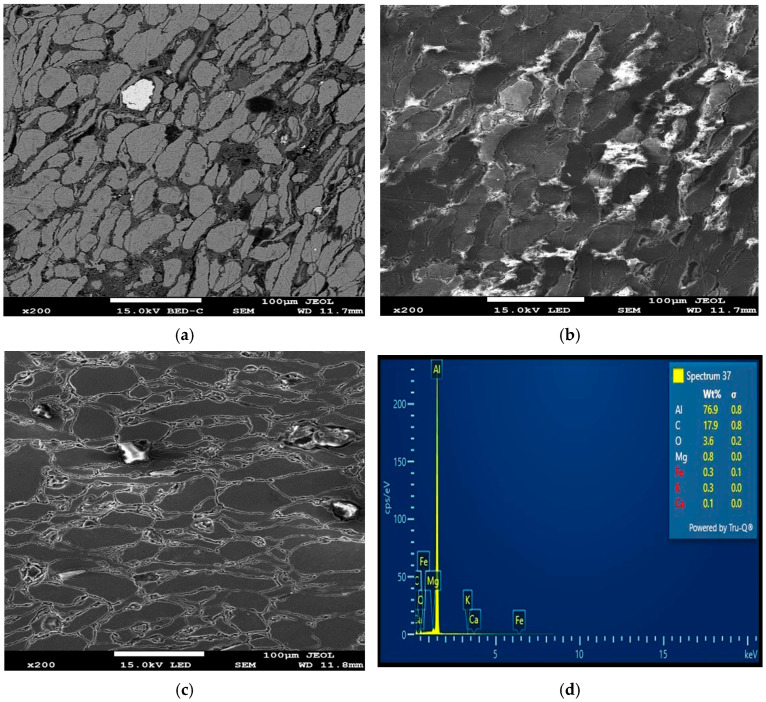
SEM images and SEM-EDS analysis of Al/Mg/PPA composites (**a**,**b**) A5 (**c**,**d**) A5.

**Figure 7:**
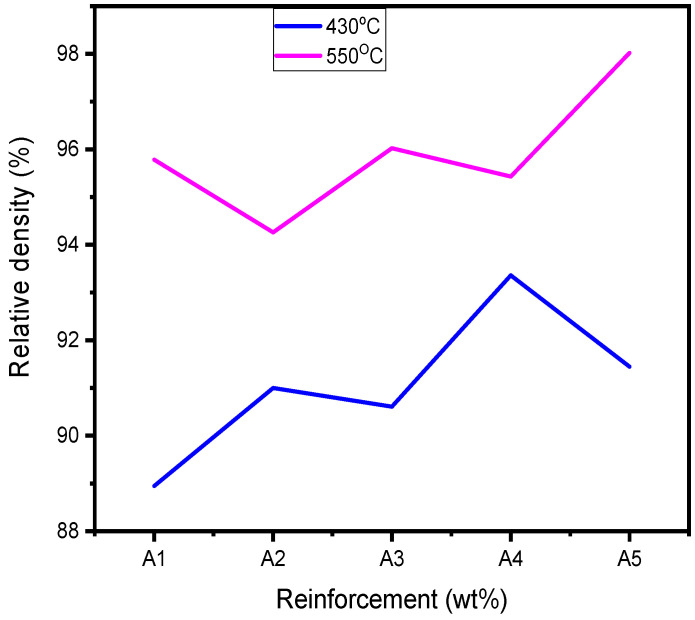
Influence of PPA reinforcement and sintering temperature on the RD of Al/Mg/PPA composites.

According to Table 3 and Figure 5, Figure 6 and Figure 7, higher sintering temperatures are required to achieve higher RDs. As seen from Table 3, the lowest sintering temperature of 430 °C produced the lowest RD (88.95%) of the sintered samples, while the highest RD of 99.05% was obtained at a sintering temperature of 520 °C. Therefore, it can be inferred that the sintering temperature is an important factor in the densification of the Al/Mg/PPA composites [23]. Powder particles are transported by diffusion. According to Equation (6), sintering AMC at high temperatures increases the particles’ thermal energy and diffusion rate [22,24]. A higher diffusion rate depends on the sintering temperature and holding (sintering) time and is essential for greater consolidation of the composites to create a denser structure. For instance, the microstructure of the composite shown in Figure 6a,b was sintered using a temperature of 430 °C. Poor grain boundaries and pores are evident. Comparing the microstructures of Figure 6a,b with the one of Figure 6c, it can be seen that the microstructural features of Figure 6c is more enhanced with improved grain boundaries and few pores. This is because the microstructure of Figure 6c is sintered at higher temperature of 520 °C. Increased porosity is a function of poor grain boundaries and an increased number of pores. This explains why the RDs of the Al/Mg/PPA samples increased with the rise in the sintering temperature, as illustrated in Figure 7. Furthermore, the SPS machine’s higher heating rate necessitated a shorter holding (sintering) time of 4 min, as indicated by Figure 4. Therefore, sintering at higher temperatures and extended holding (sintering) times may result in grain growth (coarse grains), consequently increasing porosity.

(6) $$D=D_{0}\;exp(\frac{-Q}{RT})$$

where D = diffusion coefficient, *D*_0_ = constant, Q = activation energy, R = Boltzmann constant, and T = temperature.

The compaction pressure is another important factor in the powder metallurgy process. The initial consolidation of powder particles is facilitated by compaction pressure. In SPS, the sintering temperature and compaction pressure are applied simultaneously. A certain amount of cold welding happens when the sintering pressure rises, increasing powder particle consolidation through proper neck formation. More so, the inherent friction between the powder particles and the die walls is reduced through the sintering pressure. It is challenging to achieve complete densification because friction between powder particles alters density and decreases plastic flow. However, higher densification can be achieved when compaction pressure and sintering temperature are applied concurrently because this increases the mass transport of powder particles by plastic flow and diffusion. Furthermore, it can be seen from Table 4 and Table 5 that the heating rate and holding time have a smaller impact on the RD of the composites when compared with the reinforcement material and sintering temperature. The holding time enables the composites to absorb enough thermal energy at the sintering temperature before quenching.

### 3.3. Influence of Matrix Reinforcement Characteristics and Microstructure on RD

The homogeneity of the powder particles is vital for higher densification, while powder particle agglomeration results in increased porosity. It is obvious from the optical and SEM images that the Al, Mg, and PPA particles are homogeneously distributed with little to no considerable agglomeration. This indicates that high-energy ball milling is an efficient method for achieving powder particle homogeneity [18,25].

More so, it can be deduced from Table 3 that as the weight fraction of PPA increases, the RD of the fabricated composites rises. This is a confirmation that the PPA reinforcement has a significant influence on the RD of the composites. From Table 3, sample 5 (Al/2Mg/PPA) has the highest porosity or the lowest RD, while sample 16 (Al/2Mg/15PPA) has the maximum RD or the lowest porosity. According to Equation (3), the composites’ RD rises as the theoretical densities drop. As the weight fraction of PPA increases, the theoretical densities drop, as seen in Table 2. For example, the theoretical density of sample A2 (Al/2Mg/5PPA) drops from 2.6636 g/cm^3^ to 2.6476 g/cm^3^ as the weight fraction of PPA reinforcement increases from 5 wt% to 10 wt%, and so on. When the concentration of the PPA increased from 15% to 20%, the composite’s RD decreased from 99.05% to 98.02%. The porosity of the composite may have increased slightly due to an increase in particle clustering caused by high PPA concentration.

**Figure 8:**
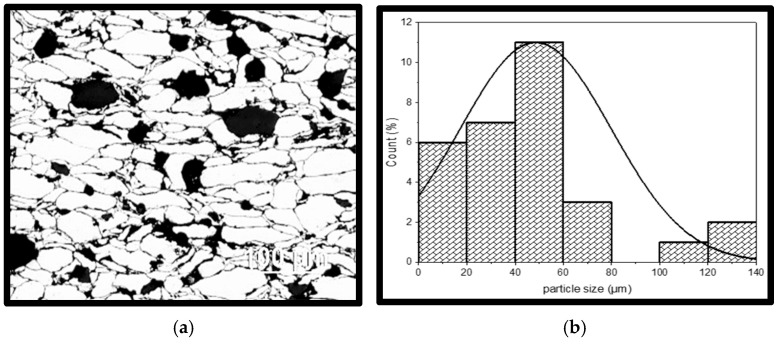

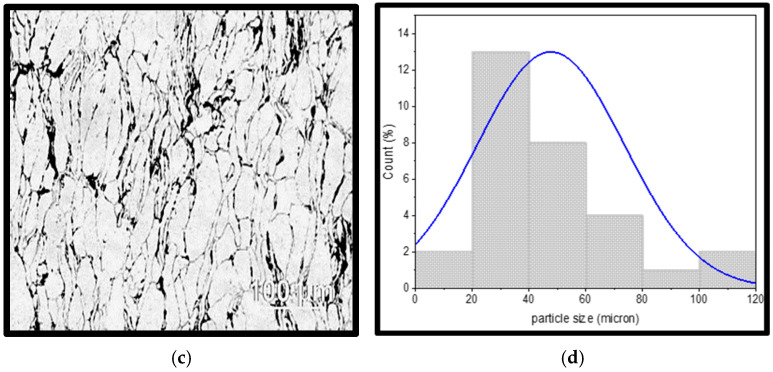
Optical micrographs and particle size distribution of sintered Al/Mg/PPA samples, (**a**,**b**) A3, (**c**,**d**) A1.

Additionally, composites densify more when fine and mixed grains are used because they pack better and have fewer pores than coarse grains. From the grain size distribution shown in Figure 8b,d using Image J software, version 1.54, it is observed that the grain sizes are mixed and range from 20 µm to 140 µm. When compared with the as-received powder particles, it can be inferred that some of the coarse grains have been fractured into finer particles through the process of ball milling and compaction. Powders with diverse particle sizes, shown in Figure 8, will minimize porosity and increase densification more so than powders with uniform particle sizes. For instance, in contrast to a combination of 20 µm (matrix) and 20 µm (reinforcement) or 120 µm (matrix) and 120 µm (reinforcement), a combination of powder particle sizes of 20 µm (matrix) and 60 µm (reinforcement) or 25 µm (matrix) and 41 µm (reinforcement) is more likely to produce higher densification. Fine and mixed grain sizes pack better, resulting in higher densification.

### 3.4. Micro-Hardness of Sintered Al-Mg-PPA Samples

**Figure 9:**
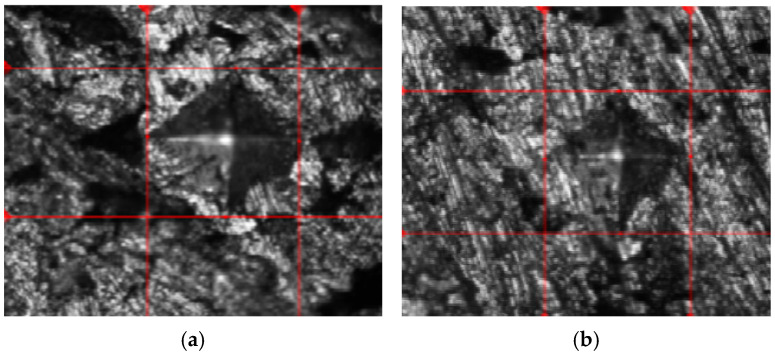
Indentation images for the hardness of sintered Al/Mg/PPA samples (**a**) first, (**b**) second image.

As earlier stated, the hardness of the fabricated Al/Mg/PPA samples was determined using a Vickers’ hardness testing machine equipped with a diamond pyramid indenter. The result of the Vickers hardness test is presented in Table 3. From the Table, the average micro-hardness of the sintered aluminum matrix is 41.2 HV, while the highest micro-hardness of the sintered composites is 77.0 HV. Figure 9a,b shows the indentation for the hardness of the composites during the hardness test. Factors such as reinforcement material characteristics and processing parameters can be attributed to the improved hardness of the Al/Mg/PPA composites [25,26].

### 3.5. Optimal Input Parameters for Micro-Hardness

**Figure 10:**
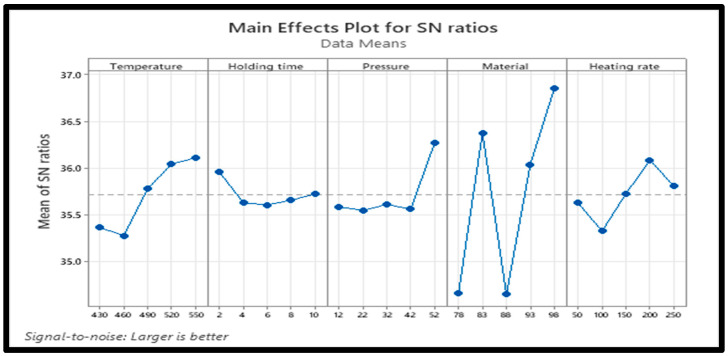
Main effects plot of signal-to-noise ratio for hardness.

The Taguchi optimization process is used to determine the optimal processing parameters that maximize the micro-hardness of the composites. Using Minitab 20 and the larger-the-better approach expressed in Equation (5), the optimization results are presented in Table 6 and Table 7 and Figure 10.

The results presented in Table 6 and Table 7 show that the material for the composites has the strongest influence on the hardness of the composites (rank 1), followed by the sintering temperature (rank 2), and so on; the holding time has the weakest influence on the hardness. The main effects plot for SN ratios shown in Figure 10 reveals that the optimal processing parameters for the best hardness of Al-Mg-PPA composites include a sintering temperature = 550 °C, holding time = 2 min, compaction pressure = 52 MPa, reinforcement concentration = 15 wt% PPA, and a heating rate = 200 °C. As evident from Table 7, the effect of material on the micro-hardness is statistically significant.

### 3.6. Influence of Sintering Parameters and Microstructure on Micro-Hardness

As illustrated in Figure 10, high sintering temperature and compaction pressure are required for the enhanced Al/Mg/PPA composites’ hardness. The sintering temperature facilitates the consolidation of the composites’ powder particles by causing interparticle welding to occur. This enhances interfacial bonding strength, increasing hardness. At higher sintering temperatures, greater consolidation of powder particles takes place, reducing porosity. However, at higher sintering temperatures, higher interfacial precipitation occurs.

**Figure 11:**
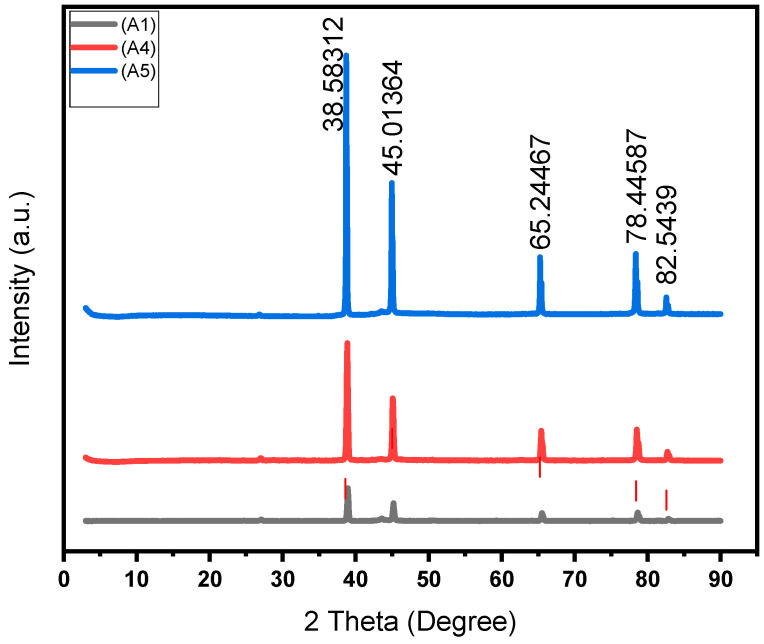
XRD pattern for the sintered Al/Mg/PPA samples.

**Figure 12:**
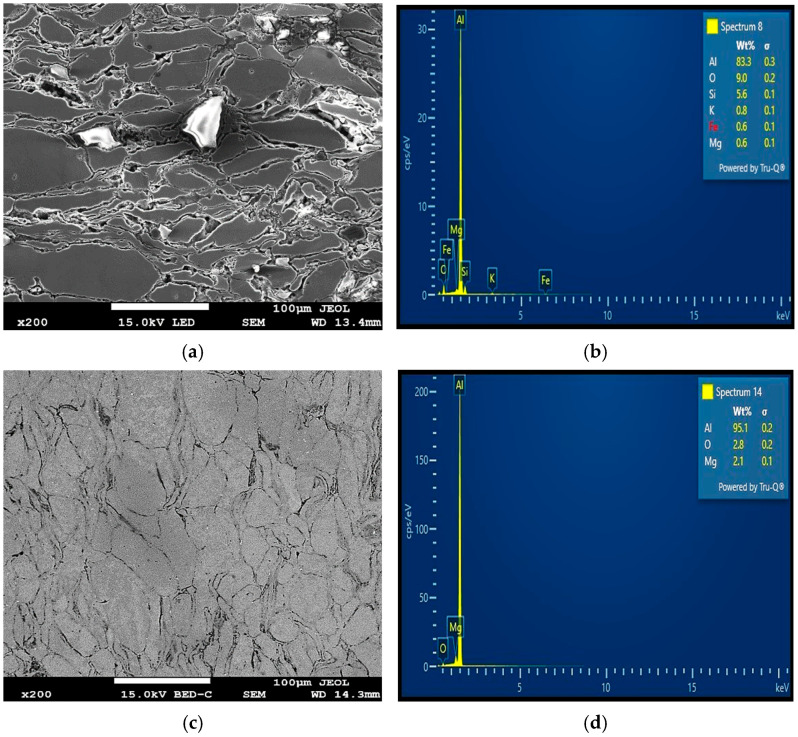
SEM micrographs and EDS analysis of Al/Mg/PPA samples (**a**,**b**) A2 and (**c**,**d**) A1.

**Figure 13:**
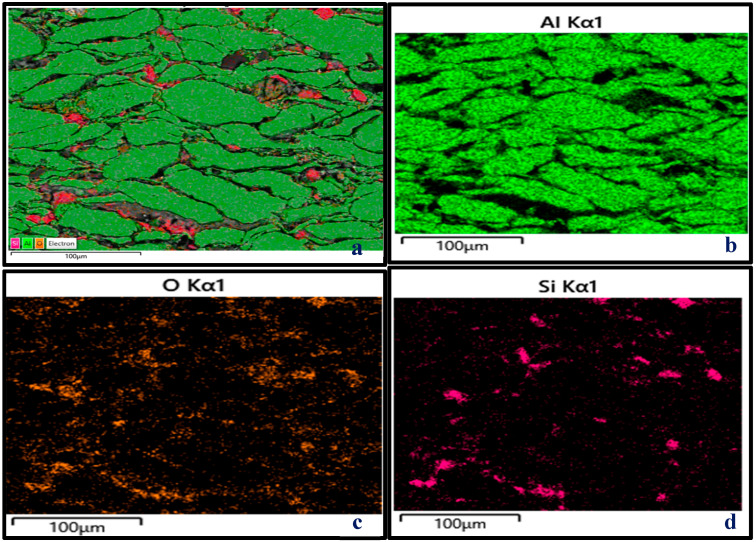
SEM mappings of Al/Mg/PPA composites (**a**–**d**) A2.

The SEM-EDS analysis, XRD peaks, and SEM mapping illustrated in Figure 6d, Figure 11, Figure 12b,d and Figure 13 show the presence of precipitates distributed within the grain boundaries. These precipitates are more in samples reinforced with PPA and, as a result, have larger areas of grain boundaries. These precipitates, which are chemically composed of elements such as Al, Mg, C, Si, K, O, and Fe, originated from the starting materials in this study. More so, aluminum easily reacts with oxygen to form oxide, which serves as impurities, hence the presence of oxygen in the samples, as shown in Figure 13. The presence of aluminum oxide in the samples may have influenced the result of the EDS analysis. The XRD pattern illustrated in Figure 11 reveals the presence of major and minor phases. Using Xpert HighScore 5.2 software for the peak analysis and from the literature, peaks such as (38.58°), (45.01°), (65.24°), (78.44°), and (82.54°) correspond to the Al, K_2_O, Al_2_O_3_, MgAl_2_O_4_, and MgO phases, respectively. Considering all the elements and compounds present in the composite samples, it can be inferred that precipitates such as AlMgSi and Al_4_C_3_ are also present in the microstructure. Interfacial precipitates such as AlMgSi and MgAl_2_O_4_ may be responsible for the improved hardness of the composites. At higher sintering temperatures, Al_4_C_3_ precipitates increase. Al_4_C_3_ is inherently brittle. While a small concentration of it can improve hardness, large concentrations in composite samples can reduce hardness [27,28]. This explains why the composite samples reinforced with 20 wt% PPA consistently showed a decline in hardness. The SPS machine uses a very high heating rate and, as a result, may not require the use of a long holding time, hence an optimal holding time of 2 min, as illustrated in Figure 10. Furthermore, the sintering pressure transmutes the loose powder into compacts during sintering. Higher pressure is required for adequate compaction, and a well-compacted sample has reduced porosity.

### 3.7. Influence of Reinforcement Characteristics and Microstructure on Micro-Hardness

**Figure 14:**
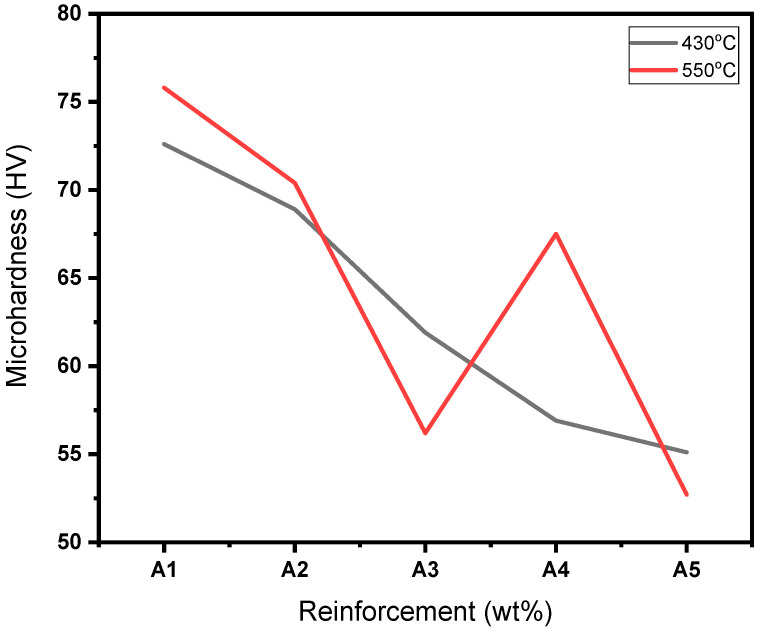
Influence of reinforcement concentration and sintering temperature on the hardness of Al/Mg/PPA composites.

As evident in Table 6 and Table 7, the reinforcement material has the greatest impact on the hardness of the Al/Mg/PPA samples. At an optimal reinforcement of 15 wt% PPA, the developed composites had the highest hardness. Beyond the 15 wt% PPA, the hardness of the composites consistently declined, as depicted in Figure 14. More so, it can be seen from Figure 10 that the highest peak for the material, which is the highest point above the dotted line, is 98 wt% Al. This represents the sintered samples without PPA reinforcement. On average, the sintered samples without the PPA reinforcement displayed the best value of hardness. This can be attributed to the stronger surface adhesion in Al/Mg samples than in Al/Mg/PPA samples. As stated earlier, the microstructure of the composites reinforced with PPA shows the presence of Al, Mg, C, Si, K, O, and Fe, as indicated by the SEM-EDS analysis in Figure 6d and Figure 12b. Similarly, the EDS analysis of the samples without PPA reinforcement shown in Figure 12d indicates the presence of Al, Mg, and O phases. This suggests that precipitates such as MgO and MgAl_2_O_4_ are present in the microstructure, as confirmed by the XRD peaks in Figure 11. While substantial precipitation of Al_4_C_3_ deteriorates the microstructure and reduces the mechanical properties such as hardness, interfacial precipitation, such as that of MgAl_2_O_4_, increases the interfacial bonding strength and hardness [29,30].

Furthermore, adding harder particles to metal matrices raises hardness. The concentration of the harder particles in the samples determines the magnitude of hardness. For instance, Tosun and Kurt [31] discovered that the hardness of AMC reinforced with SiC was higher than the ones reinforced with Al_2_O_3_. This is so because SiC (2350 HV) is harder than Al_2_O_3_ (880 HV). Therefore, adding PPA and Mg particles to the Al matrix enhanced the hardness of the fabricated samples.

The degree of wettability between the matrix and reinforcement influences hardness. To improve the wettability in this study, 2 wt% Mg was introduced into the Al/PPA mixes. A maximum hardness of 77 HV was achieved at 15 wt% PPA concentration, as seen in Table 3. However, the composites synthesized with 20 wt% PPA (A5) consistently showed a decline in hardness, as illustrated in Figure 14. This can be explained by the weak interfacial bonding strength and poor surface adhesion. Therefore, it can be inferred that as the concentration of PPA increases, the degree of wettability and surface adhesion drops. In addition, the hardness of Al/Mg/PPA composites reinforced with 20 wt% PPA dropped because the PPA particles may not be as hard as the synthetic reinforcement particles such as B_4_C and SiC. More so, the K_2_O phase in the microstructure, as indicated by the XRD peaks, may be one of the weak phases that can impair hardness. Therefore, hardness reduces as the weight fraction of the weak phase increases in the composites.

The homogeneity of the powder particles, as indicated by the SEM and optical images, is due to the improved hardness of the composites. Particle agglomeration results in micro-cracks, weak interfacial bonding, and increased pores, which impair hardness. At higher concentrations of PPA, particle clustering is inevitable, thereby reducing hardness.

The refinement of the matrix and reinforcement particles significantly influenced hardness. Fine particles enhance hardness compared to coarse particles, and mixed particle sizes promote better packing than uniform-size particles [32,33]. The improved hardness of Al/Mg/PPA composites can be attributed to the fine and mixed particle sizes, as shown in Figure 8. During milling, the high-energy balls collide with the powder particles, thereby fracturing larger particles into fine ones. In addition, the plastic deformation that occurs during the ball milling process causes strain hardening and particle transmutation. Fine and diverse particle sizes result in better particle packing and alignment, making it tougher for dislocation motion to pass through. This ultimately enhances hardness.

## 4. Conclusions

This article reported an investigation of the densification and hardness properties of Al/Mg/PPA samples with reference to PPA reinforcement and SPS parameters. The results show that the enhanced densification and hardness of the synthesized composites are caused by factors such as processing parameters, reinforcement characteristics, and particle surface adhesion. As the sintering temperatures increased, the porosity of the sintered samples decreased. The RD was significantly impacted by the sintering temperature and material, whereas the matrix/reinforcement characteristics significantly impacted the hardness property. The best RD of Al/Mg/PPA composites was obtained at 550 °C sintering temperature, 4 min holding time, 42 MPa compaction pressure, 250 °C heating rate, and 15 wt% PPA reinforcement. The values of these parameters for the optimal hardness were 550 °C, 2 min, 52 MPa, 200 °C, and 15 wt%, respectively.

## Figures and Tables

**Table 1:** Sample designation and their theoretical densities.

Sample Designation	Composition	Theoretical Density (g/cm^3^)
A1	98Al:2Mg	2.6816
A2	93Al:2Mg:5PPA	2.6636
A3	88Al:2Mg:10PPA	2.6476
A4	83Al:2Mg:15PPA	2.6276
A5	78Al:2Mg:20PPA	2.6096

**Table 2:** Sintering parameters for Al/Mg/PPA composites.

Parameters	Levels				
	1	2	3	4	5
Sintering temperature (ST) °C	430	460	490	520	550
Holding (sintering) time (HT) min	2	4	6	8	10
Sintering pressure (SP) MPa	12	22	32	42	52
Heating rate (HR) °C/min	50	100	150	200	250

**Table 3:** RD and hardness results of Al/Mg/PPA samples.

Exp. Runs	Input Processing Parameters					Results			
	ST (°C)	HT (min)	‘SP’ (MPa)	PPA wt%	HR (°C/min)	Av. Exp. Density (g)	RD (%)	Average Hardness (HV)	Standard Dev. for Hardness
	Experimental Values								
**1**	430	2	12	20	50	2.3864	91.45	55.1	7.1
**2**	430	4	22	15	100	2.4531	93.36	56.9	3.8
**3**	430	6	32	10	150	2.3989	90.61	61.9	7.6
**4**	430	8	42	5	200	2.4250	91.00	68.9	4.6
**5**	430	10	52	0	250	2.3853	88.95	72.6	7.3
**6**	460	2	22	10	200	2.4660	93.14	54.5	7.0
**7**	460	4	32	5	250	2.5380	95.30	63.0	7.9
**8**	460	6	42	0	50	2.5021	93.31	61.5	1.6
**9**	460	8	52	20	100	2.4323	93.21	53.3	2.5
**10**	460	10	12	15	150	2.5280	96.21	58.8	5.3
**11**	490	2	32	0	100	2.4644	91.90	64.7	4.4
**12**	490	4	42	20	150	2.4592	94.24	53.5	4.5
**13**	490	6	52	15	200	2.4759	94.23	77.0	6.2
**14**	490	8	12	10	250	2.4750	93.48	52.1	7.3
**15**	490	10	22	5	50	2.4938	93.63	63.5	5.9
**16**	520	2	42	15	250	2.6026	99.05	71.5	7.0
**17**	520	4	52	10	50	2.5243	95.34	55.7	5.5
**18**	520	6	12	5	100	2.5159	94.45	61.7	5.4
**19**	520	8	22	0	150	2.5059	93.45	74.5	3.2
**20**	520	10	32	20	200	2.5168	96.44	56.1	6.2
**21**	550	2	52	5	150	2.5107	94.26	70.4	8.7
**22**	550	4	12	0	200	2.5685	95.78	75.8	4.9
**23**	550	6	22	20	250	2.5580	98.02	52.7	3.1
**24**	550	8	32	15	50	2.5074	95.43	67.5	4.8
**25**	550	10	42	10	100	2.5421	96.02	56.2	4.5
**Al**	550	4	12	100	200	2.6901	99.63	41.2	3.3

**Table 4:** Response for signal-to-noise (SN) ratios for RD.

Level	Temperature	Holding Time	Pressure	Material	Heating Rate
1	39.19	39.46	39.49	39.52	39.45
2	39.48	39.54	39.49	39.61	39.44
3	39.42	39.47	39.45	39.43	39.44
4	39.62	39.40	39.53	39.44	39.47
5	39.64	39.48	39.39	39.34	39.54
Delta	0.45	0.14	0.14	0.28	0.11
Rank	1	4	3	2	5

**Table 5:** Analysis of Variance for RD.

Source	DF	Adj SS	Adj MS	F-Value	*p*-Value
Regression	5	85.147	17.029	8.05	0.000
Temperature	1	62.362	62.362	29.49	0.000
Holding time	1	0.414	0.414	0.20	0.663
Pressure	1	1.528	1.528	0.72	0.406
Material	1	17.500	17.500	8.28	0.010
Heating rate	1	3.344	3.344	1.58	0.224
Error	19	40.175	2.115		
Total	24	125.322			

**Table 6:** Signal-to-noise ratios of micro-hardness (larger is better).

Level	Temperature	Holding Time	Pressure	Material	Heating Rate
1	35.37	35.96	35.59	34.67	35.63
2	35.28	35.63	35.55	36.38	35.33
3	35.78	35.61	35.61	34.66	35.73
4	36.05	35.66	35.57	36.04	36.09
5	36.11	35.73	36.27	36.85	35.81
Delta	0.83	0.35	0.72	2.19	0.76
Rank	2	5	4	1	3

**Table 7:** ANOVA for micro-hardness.

Source	DF	Adj SS	Adj MS	F-Value	*p*-Value
Regression	5	643.95	128.789	2.63	0.057
Temperature	1	137.12	137.117	2.80	0.111
Holding time	1	5.51	5.511	0.11	0.741
Pressure	1	51.01	51.005	1.04	0.320
Material	1	406.70	406.695	8.31	0.010
Heating rate	1	43.62	43.618	0.89	0.357
Error	19	929.86	48.940		
Total	24	1573.81			

## Data Availability

The original contributions presented in this study are included in the article. Further inquiries can be directed to the corresponding author.
